# Qualitative evaluation of the general practitioner chronic non-communicable diseases training programme

**DOI:** 10.1186/s12909-020-02226-x

**Published:** 2020-09-10

**Authors:** Chunyu Zhang, Fang Fang, Mingqiang Peng, Ying Zhao, Ruixue Liu, Cunbo Jia

**Affiliations:** 1 Department of Health Reform and Development, China-Japan Friendship Hospital, Yinghua East Road 2#, Chaoyang District, Beijing, 100029 P.R. China; 2 Hospital Office, China-Japan Friendship Hospital, Beijing, P.R. China; 3 School of Management, Beijing University of Chinese Medicine, Beijing, P.R. China

**Keywords:** General practitioner, Continued medical education, Chronic non-communicable diseases

## Abstract

**Background:**

In China, general practitioners have limited ability to provide care for common chronic non-communicable diseases because they lack postgraduate training. In an attempt to improve general practitioners’ skills in this regard, the present authors previously launched the Chronic Non-Communicable Diseases Training Programme. The present study aims to evaluate the effectiveness of this programme.

**Methods:**

Thirty-nine trainee general practitioners who participated in the programme underwent semi-structured interviews, which explored how they performed the training, what they achieved from the programme, and their suggestions for future programmes. The interview data were analysed using a thematic analysis approach.

**Results:**

Under the guidance of supervisors, the thirty-nine trainee general practitioners completed the structured but individualised training plan, which comprised a four-day basic theory class, 3 months practising in a ward, and 6 months assisting in an outpatient clinic. They reported an improvement in their ability to provide care for chronic non-communicable diseases and perform two-way referral, as well as their communication with patients. They also reported that, since returning to their communities, they had become more confident, were building better relationships with their patients, and had changed their clinic behaviours from copying prescriptions to making medical decisions independently. Their principal suggestion for the training programme was to alter the order of the training, as they preferred to practice in the ward before assisting in the outpatient clinic.

**Conclusion:**

The course comprised a learner-centred, practice- and apprenticeship-based, general-practitioner training programme. Given the participants’ progress and the beneficial effects of the programme reported in the interview data, it appears to be worthwhile to extend the General Practitioner Chronic Non-Communicable Diseases Training Programme.

## Background

Chronic obstructive pulmonary disease (COPD), diabetes, stroke, and hypertension are the most important causes of morbidity and mortality in the Chinese community [1–4]. As a result, general practitioners (GPs), who are generally the first point of access to the health-care system, should be proficient at managing these four common, chronic, non-communicable diseases [5, 6]. Over the past decade, the World Health Organization (WHO) and countries such as Norway and the US have given high priority to strengthening GPs’ skills [7–9]. However, in China, most GPs have not received specific postgraduate or continuing medical education and training focussed on developing the skills to provide care for chronic diseases. As a result of this lack of training, GPs in China have limited ability to diagnose and treat these four common chronic diseases [10–12].

To help improve GPs’ skills, the present authors launched, with the support of local health authorities, the Chronic Non-Communicable Disease Training Programme [13]. This programme, based on an apprenticeship model, comprised a course that features intensive clinical training and basic theory, as well as attendance at an outpatient clinic over a period of 6 months and 3 months of inpatient practice. First, the participating GPs chose one of four diseases (COPD, diabetes, stroke, or hypertension) as their training field depending on their own interests and the demands of their communities. The trainee GPs then practised in a tertiary hospital (the leading tertiary hospital in the local medical group) under the guidance of supervisors from April 1st to December 31st, 2019. All of the supervisors were specialists selected from the tertiary hospital. The present study aims to investigate the effectiveness of this programme and provide suggestions for future training programs. Specifically, we seek to answer the following questions: What skills did the trainee GPs obtain? Did their ability to provide care for the four common chronic non-communicable diseases improve? Did their work as a community GP improve as a result of attending the training program?

## Methods

This research employed a descriptive, interpretive qualitative study design involving thematic analysis to provide a greater depth of understanding regarding the GPs’ experiences [14].

### Sampling and recruitment

One month after the programme finished, all of the 39 trainees were invited, via a short telephone message, to participate in a 30-min, face-to-face, semi-structured interview, for which their data would remain anonymous.

### Interviews

We conducted face-to-face interviews, which enabled the researcher to observe the subjects’ body language and nonverbal cues in January 2020. This allowed us to probe the interviewees’ real thoughts and judge the reliability of their responses. To maintain anonymity, all interviewees were subsequently assigned a code prior to the review of the interview data. Field notes were taken during and immediately after each interview, and each interview was audio-recorded. The interviews were held in private rooms because we felt that the participants would be more comfortable in such an environment and would consequently provide more open answers.

### Analysis

Audio recordings of the interviews were transcribed using Xunfeiyuji (V4.9.7.1432) [15]. Thematic analysis initially involved a thorough reading of each transcript by two members of the research team, which resulted in the generation of initial codes that were subsequently discussed at a project meeting. NVivo software version 11 (QSR International) [16] was used to organise and code the data. After all of the data were initially coded, overarching themes were generated by combining codes. Themes were then reviewed and refined, and linkages between themes were established.

## Results

Overall, 39 GPs participated in this study; of these, 33 were female (84.6%), and six were male (15.4%). The average age was 35.05 ± 5.28 years, with a range of 27 to 47. In this programme, 12, 12, eight, and seven GPs studied COPD, diabetes, stroke, and hypertension care, respectively. Of these, 31 (79.5%) had bachelor’s degrees, and 24 (61.5%) had a medium technical rank. As a result of the respective availabilities of the supervisors, 14 of the GPs had initially assisted their supervisors’ outpatient consultations and then practised in the ward, seven GPs had received this training in the reverse order, and 18 GPs had initially assisted the outpatient consultations for a shorter period, then practised in the ward, and then performed the remainder of their outpatient consultant assistance. Although their training orders differed, the total training length was the same for all GPs (9 months), comprising 3 months in a ward and 6 months in an outpatient clinic (see Table 1).

**Table 1:** The trainees’ characteristics

Training field	COPD	Diabetes	Stoke	Hypertension
Gender				
Male	4 (33.3%)	2(16.7%)	0(0%)	0(0%)
Female	8(66.7%)	10(83.3%)	8(100%)	7(100%)
Age (years)				
< 30	2(16.7%)	1(8.3%)	3(37.5%)	1(14.3%)
30–39	8(66.7%)	8(66.7%)	4(50.0%)	6(85.7%)
≥ 40	2(16.7%)	3(25.0%)	1(12.5%)	0(0%)
Work year				
≤ 5	3(25.0%)	2(16.7%)	4(50.0%)	2(28.6%)
6–10	2(16.7%)	1(8.3%)	1(12.5%)	2(28.6%)
> 10	7(58.3%)	9(75.0%)	3(37.5%)	3(42.9%)
Education background				
Bachelor’s	9(75.0%)	10(83.3%)	6(75.0%)	6(85.7%)
Master’s	2(16.7%)	2(16.7%)	2(25.0%)	1(14.3%)
Doctorate	1(8.3%)	0(0%)	0(0%)	0(0%)
Technical rank				
Junior	3(25.0%)	4(33.3%)	3(37.5%)	3(42.9%)
Medium	9(75.0%)	7(58.3%)	4(50.0%)	4(51.7%)
Senior	0(0%)	1(8.3%)	1(12.5%)	0(0%)
Training model				
O-I	4(33.3%)	3(25.0%)	2(25.0%)	5(71.4%)
I-O	5(41.7%)	1(8.3%)	0(0%)	1(14.3%)
O-I-O	3(25.0%)	8(66.7%)	6(66.7%)	1(14.3%)
Total	12	12	8	7

O-I: Initially assisting the outpatient consultant, and then practising in the ward

I-O: Initially practising in the ward, and then assisting the outpatient consultant

O-I-O: Initially assisting the outpatient consultant, then practising in the ward, and finally assisting the outpatient consultant again

*COPD* Chronic obstructive pulmonary disease

Interview length ranged from 20 to 50 min, and the average duration was 30 min. Five themes emerged through analysis of the semi-structured interviews. Of these themes, two related to the training pattern, three related to acquiring skills, and one concerned a suggestion for improving the course (see Table 2).

**Table 2:** Themes identified

**Training pattern**	
1. Type of training the trainee GPs received	
2. Support the trainee GPs received from their supervisors	
**Acquiring skills**	
3. What the trainee GPs learned from the programme	
4. The current work benefits from the trainee GPs’ training experience	
**Suggestion**	
5. Suggestions for improving the programme	

### Theme 1: type of training the trainee GPs received

The full training course comprised three parts: a basic theory class, 3 months practising in the ward, and 6 months assisting the supervisor consultant in the outpatient clinic.

At the beginning of this programme, a four-day class regarding COPD, diabetes, stroke, and hypertension was held. The content of this basic theory class, which conformed with the textbook used in the medical university, ranged from pathogenesis, through diagnosis, to treatment.

*After graduation, I seldom opened the textbook because I was too busy. Attending the four-day class allowed me to review the information I had learned in university…*

When practising in the ward, the trainee GPs usually provided care for 2–3 patients simultaneously, like interns, while receiving guidance from their supervisors. During this period, advanced medical knowledge classes or case discussions were conducted once a week, which helped the GPs achieve their training goals.

*Supervisor Y provided me with an opportunity to share a case. My preparation for the PowerPoint presentation involved reviewing the relevant information. As a result of my preparation and presentation, I developed a deeper understanding of the case.*

In the outpatient clinic, the trainee GPs observed the methods by which their supervisors handled the outpatients. Meanwhile, the supervisors explained interesting cases to the GPs and answered their questions, which served to improve their skills.

*In accordance with the programme syllabus, every Monday afternoon and Wednesday morning another trainee and I sat next to our supervisor and managed the outpatients together. My supervisor asked the patients questions and gave suggestions; meanwhile, we wrote medical records and typed prescriptions. Sometimes, we asked questions, and the supervisor gave explanations during the intervals between outpatients.*

### Theme 2: support the trainee GPs received from their supervisors

The supervisors’ main responsibilities were imparting medical knowledge and ensuring that the trainee GPs exercised safe medical behaviour throughout the entire training period. Meanwhile, the supervisors also made special arrangements for every GP based on the GPs’ respective abilities and future goals.

*Supervisor X always allocated simple cases to me because my knowledge foundation was relatively weak, and I had less experience in the ward. At first, she checked every treatment plan I made; later, I was able to provide simple, common medical advice independently.*

The supervisors also readily answered the trainee GPs’ questions.

*If I had a question, I could immediately ask my supervisor…Sometimes, he was too busy to answer immediately, but he would eventually answer, no matter how late it was. Although I have now returned to the community health care centre, whenever I have a question, I can still immediately consult my supervisor.*

### Theme 3: what the trainee GPs learned from the programme

All of the trainee GPs reported that they had acquired comprehensive and advanced skills.

*In the past, there was no insulin in our health community centre because of the fear of causing adverse consequences as a result of inaccurate prescription. After receiving training, however, I have become confident in my ability to prescribe insulin. Further, I now know how to adjust its dose to suit patients’ individual situations. With the support of the head of my community health care centre, insulin has now been introduced in our centre, and diabetes patients can now refill their insulin prescriptions here.*

*In the training programme, I learned for the first time that the pulmonary function test is the gold standard for diagnosing COPD; there are many COPD patients in my community. After spending one month practising in the pulmonary function test department, I can now not only operate the lung function meter correctly, but also interpret lung function test reports.*

Thirty-seven of the 39 GP trainees reported that, as a result of the training, they had learned to handle some complex cases independently.

*Refractory hypertension is not uncommon in my community, but before training, I was confused about it and referred patients with this condition directly to a tertiary hospital. Since the training, however, my recent treatment plans for treating refractory hypertension have all been approved by my supervisor.*

Ten of the trainee GPs mentioned that they had learned how to communicate with patients as a result of observing their supervisors.

*My supervisor, Doctor S, usually informs her patients, in plain language, about the extent of their respective illnesses and why they need to be managed. When a patient does not understand, she uses real-life examples to provide explanations. Since returning to my community, I have adopted her method of communicating with patients, and the patients have consequently shown their satisfaction with me.*

The majority of the GPs reported that they had made progress in relation to performing differential diagnosis.

*When practising in the ward, I regularly encountered patients with illnesses other than the four targeted diseases. For example, interstitial lung disease is a rare disease in my community, but the experience of learning how to diagnose and treat this disease has broadened my mind and helped me diagnose espiratory diseases such as COPD more confidently.*

All of the GPs confirmed that their clinic behaviour had changed from copying prescriptions to making carefully considered medical decisions and that their basic diagnosis ability had improved.

*I used to prescribe medicine by copying the prescriptions provided by patients; this was because I had a relatively poor ability to determine patients’ requirements. The patient who left just now has had a cough for half a year; I performed a pulmonary function test for him and found that his FEV1/FVC is less than 70%. After that, I prescribed some inhaled medication (during the interview, the GP showed us the patient’s pulmonary function result and explained why he had prescribed inhaled medication).*

### Theme 4: the current work benefits from the trainee GPs’ training experience

Firstly, all of the trainee GPs reported becoming more confident as a result of improvements in both their ability to use clinical technology and their communication skills.

*As patients could not undergo CT or MRI examinations at our (community health) centre, before the training I did not know how to read patients’ CT or MRI films. After returning to my community centre after the training programme, I began to ask patients to bring their CT or MRI films with them. At first, I could see the doubt in their eyes. I explained the results of the films to them and gave my diagnosis and advice. Eventually, more and more patients began to believe me. One patient told me: ‘Doctor X, your explanation was the same as that given to me by the tertiary hospital physicians’. Now, I insist that patients bring their previous examination results with them.*

Second, a two-way referral channel was established between the trainee GPs and their supervisors as a result of the good relationships they had fostered during the programme. Consequently, patients were able to gain access to a continuous health-care service referral system.

*Quite a few patients heard I had been trained in the China-Japan Friendship Hospital, and came to visit me. There was a 60-year-old man who, after asking for his symptom and disease history, I thought had interstitial lung disease; however, I was not 100% sure because some examination results were not available. I asked him to visit my supervisor, and the diagnosis was the same as I had suspected. Now, the patient visits me routinely to refill his medication or adjust his treatment plan.*

Finally, the relationship between GPs and their patients also improved as a result of this programme. The GPs’ improved health-care abilities caused patients to develop greater trust in their GPs and to show higher willingness to visit community health care centres.

*Before training, I knew that aminophylline and glucocorticoids were administered to COPD patients, but I did not know why or how to use them. As a result, I only copied patients’ existing prescriptions and was afraid to change dosages. In this programme, I systematically learned how to diagnose and treat COPD. The more knowledge I acquired, the more clearly I could provide explanations to patients. I explained in detail why they should use this medication and why they should increase or decrease the dosage. The patients always felt satisfied with my explanations and were willing to visit me again.*

### Theme 5: suggestions for improving the program

Seven of the 14 trainee GPs who had initially assisted in the outpatient clinic and then practised in the ward suggested that it is better to practice in the ward before assisting in the outpatient clinic.

*When I was in the outpatient department, I regularly felt confused because of my poor knowledge foundation. There were even some patients with diseases I had never heard of, so the knowledge I gained was limited. However, practising in the ward is a good opportunity to absorb a lot of knowledge in a short time. I believe that I would have achieved more if I had worked in the ward first and then attended the outpatient clinic.*

Thirty-five of the trainee GPs suggested extending the time in the ward and expanding the targeted diseases.

*Three months practising in the ward is not enough to greatly improve one’s skills. I needed the first month to acquaint myself with the hospital information system and the rules of a tertiary hospital.*

*In my community, besides those with COPD, there are many patients with cardiovascular disease. Thus, if there is an opportunity, I hope to also receive training in a department of cardiology.*

## Discussion

Although China has made remarkable progress in regard to strengthening its primary health-care system, GPs, as the gatekeepers to the health-care system, still lack the knowledge to diagnose and treat common chronic non-communicable diseases [17]. Further, there is little continuity and pertinence in the practical training currently provided for GPs [17, 18]. As a result of these deficiencies, patients have long been reluctant to visit community health care centres, instead they visit secondary or tertiary hospitals, which have, consequently, become crowded [19, 20]. This situation means that the majority of initial diagnoses are implemented at secondary or tertiary hospitals and that GPs’ main task is, consequently, to copy patients’ prescriptions when they seek a refill [21, 22]. Our Chronic Non-Communicable Diseases Training Programme is a learner-centred approach designed to resolve this problem. Although we have no quantitative data to evaluate the effectiveness of the programme, we found through qualitative interviews that the trainee GPs who received this training felt that they had greatly improved their disease diagnosis and treatment skills, communication with patients, confidence, and clinical practice habits.

All of the above achievements are a result of the application in the training programme of the apprenticeship model, which is widely used in the education field because it involves supervisor responsibility and increases motivation to participate [23, 24]. As part of the programme requirements, the supervisors developed individualised training plans for each trainee GP. As we learned from the interviews, the supervisors provided both active and passive support. For example, supervisors arranged for GPs who had a weaker knowledge base to care for inpatients who had relatively less serious conditions. In our interviews, all of the trainee GPs agreed that, when they encountered difficulties, their supervisors helped them to resolve the problems in a timely fashion. Further, as a result of their good relationship, supervisors continued to check the GPs’ analyses and diagnoses even after the GPs had returned to their communities. Gradually, under the guidance of the supervisors, the GPs acquired more skills, became more confident, and became more willing to learn.

The training programme was learner-oriented, and it can be considered that this approach produces better results than a non-learner-centred model, as this has been reported in previous studies [25]. The head supervisors for each speciality had experience working as vice-directors of health community centres for at least 1 year, or of performing regular outreach work in communities. Thus, although the supervisors were from tertiary hospitals, they were familiar with the situation of community health care centres and the needs of such patients. Therefore, both the overall and the individual training plan were designed to closely meet GPs’ needs and to allow the GPs to continue to apply the knowledge acquired once they had returned to their communities.

This was also a practice-based learning program; an approach that is strongly endorsed by medical experts, and that has previously been proven effective for GP postgraduate training [26]. During the three-month practice in the ward and the 6 months assisting in the outpatient clinic, the trainee GPs frequently practised new skills such as reading CT results, performing pulmonary function tests, and administering insulin; thus, they gradually acquired these necessary skills. This is consistent with recommendations by the WHO and many governments, who regard strengthening primary health care as an effective method of enhancing chronic care [27–29]. We also believe that, if an increasing number of GPs receive practice such as this, many routine medical checks and treatments that are currently performed by specialists in tertiary hospitals could instead be performed by GPs in the near future.

Patients will be the ultimate beneficiaries of this programme. It is a common phenomenon in China for patients to be reluctant to consult GPs because they lack skills in chronic disease care [19, 30]. After receiving this training programme, the GPs reported gaining the ability to resolve minor health problems in their communities. As similar studies have found, such training programs provide several benefits for patients; for example, high-level GP consultation is associated with less travel time for patients [31]. Additionally, a smooth two-way referral was created after the training program; this is important because the existence of well-informed GPs and specialists improves patients’ access to continuous health care, and removes undue waiting times for appointments.

The use of semi-structured interviews, the arrangement of private interview rooms, the maintenance of anonymity, and the involvement of researchers from outside the programme contributed to ensuring the veracity of the GPs’ responses. Regarding the trainee GPs’ achievements, there was little observation of actual practice, meaning the trainee GPs’ reports could not be confirmed. Consequently, there is a need for further empirical research to examine the effectiveness of the programme. Meanwhile, the ratio of female trainee GPs to their counterpart (5.5) is higher than the general trend (1.97). Although we found that there was no difference between genders for training effectiveness, we still could not determine whether female trainee GPs were more eager to learn or whether gender was associated with the training effectiveness. Quantitative study is needed to answer these questions.

## Conclusions

The programme improved the GPs’ ability to provide care for four common chronic non-communicable diseases. The programme was particularly successful for creating connections between GPs and specialists and effecting an improvement in primary care resources for common chronic diseases in communities. We hope this programme will lead to improved patient care and feel that our findings could offer lessons for other countries and regions.

## Data Availability

The datasets used and/or analysed during the current study are available from the corresponding author on reasonable request.

## Abbreviations

COPD: Chronic obstructive pulmonary disease
GP: General practitioner
WHO: World Health Organization
